# A cross-sectional study of variations in schoolwork stress in academic upper secondary school classes in Mid-Norway

**DOI:** 10.1177/14034948241242939

**Published:** 2024-04-10

**Authors:** Vegard Johansen, Ingvild Røsand

**Affiliations:** Norwegian University of Science and Technology, Trondheim, Norway

**Keywords:** schoolwork stress, adolescent health, mental health, secondary schools, survey, multilevel modeling, school class effect

## Abstract

**Aims:**

This paper investigates stress related to schoolwork among students in academic upper secondary schools. The research questions asked are: 1. To what degree does students’ schoolwork stress vary between academic classes?; And 2. are perceptions of classroom goal orientation, academic achievement, sex and parental education related to schoolwork stress?

**Methods:**

A cross-sectional survey was done in the final year of upper secondary school in 71 school classes from 13 schools. A total of 1955 students in academic education programs were invited to participate in the survey, and 1511 completed the survey; the response rate was 77%. The outcome measure was a composite measure of schoolwork stress (alpha = 0.81). Multilevel modeling was used to estimate school class-level effects.

**Results:**

The mean value of schoolwork stress was 4.0, on a scale of 1 (very little schoolwork stress) to 6 (very high schoolwork stress). About half of the students reported a score of 4 or higher. The analysis showed that individual characteristics explained most of the variation in schoolwork stress. Girls experienced a much higher level of schoolwork stress than boys (mean values of 4.3 and 3.6, respectively). There was also a significant class-level effect, estimated to 6% of the variance. Students’ perceptions of classroom goal orientation was also associated with schoolwork stress.

**Conclusions:**

**The main contribution was the discovery of significant variations in schoolwork stress between school classes. We also found that higher mastery climate was linked to lower schoolwork stress, whereas higher performance climate was linked to higher schoolwork stress.**

## Background

Several studies conducted in different countries have shown that school stress is a significant issue for many adolescents [1–4]. These studies have found that a large percentage of students experience stress related to day-to-day academic demands, such as homework assignments, class projects, tests and maintaining good grades. In addition to academic demands, adolescents might experience school stress because of their expectations about academic achievements, concerns about their future education and career prospects, and social pressure from teachers, family members and peers [1,4–5]. There is evidence to suggest that the prevalence of school stress among adolescents has increased in recent years, especially among older adolescents and girls [1,4–7].

According to Lazarus and Folkman, when individuals perceive that the demands placed on them from external factors are greater than their perceived ability to cope with those demands, they experience stress [8]. Their transactional model of stress and coping posits two important phases that are (a) cognitive appraisals that are divided in primary and secondary appraisals and (b) coping. In primary appraisal, an individual assesses what the stressor or situation means and how it can influence her, and then the person categorizes the situation as being a threat, harm, or challenge. In the school context, some students might perceive their schoolwork as challenging and associated with eagerness and motivation for learning, but other students might perceive schoolwork as threatening and leading to feelings of worry about their academic achievements. During the secondary appraisal, individuals assess their resources and capabilities to cope with the situation they perceive as stressful. What may be stressful for one student may not hold the same significance for another. In the final phase, the use of problem-focused and emotion-focussed coping strategies determine how well the individual manages the stressful situation [8].

Stress can manifest in a combination of emotional symptoms such as anxiety and feeling overwhelmed and psychosomatic symptoms such as headaches and stomach aches [1,9]. Prior studies have confirmed links between these health symptoms and central aspects of the school environment such as academic workload, competition for grades and classroom goal orientation as well as other psychosocial aspects [2,9,10].

The negative effects of school stress on academic achievements, mental health, and wellbeing are well documented. High levels of school stress are connected to academic underperformance, decreased student motivation, and dropout [3,6,11,12]. Some studies have shown that high levels of school stress are linked to mental health problems such as emotional problems, anxiety and depression [2–4,13], whereas other studies show negative relations between school stress and sleep quality, physical activity and physical health, relationships and overall quality of life [2,4,13,14].

School stress is generally perceived as a negative phenomenon, but there can also be *eustress*, i.e. stress that leads to a positive response. Studies show that experiencing school stress can help students develop better time management skills and learn to prioritise tasks [15–17], that (a moderate) level of school stress can help to increase motivation and achievement [3,6] and that experiencing and coping with school stress can build resilience and prepare adolescents for future challenges [3,13,18].

Several studies that investigated demographic factors associated with perceived school stress have found that the level of school stress increases with age, whereas results are mixed with regard to connections between parental education and occupation [6,7,18]. Adolescent girls report higher levels of school stress than boys, and it seems that girls are more exposed to the negative effects of stress [3,7,19,20]. To explain sex differences as regards school stress, it has been argued that girls undergo more pubertal changes than boys, that girls emphasise social demands to a greater extent than boys and that girls tend to place greater importance on academic achievement and may have higher education expectations [13,21–23]. It also seems that girls influence each other and exaggerate school stress to a greater extent than boys [21,22], in particular if there is a culture of performance in the school and the class [7,21,22,24].

Studies have also investigated connections between school stress and school factors such as teacher relationships, peer relationships, school effort and prior academic achievements [6,7,21,25]. Adolescents in academic programs reported higher levels of school stress compared with students in vocational or technical programs [25]. This may be due to higher workload, more competition for top grades and acceptance into universities, and high expectations from themselves, parents, teachers and peers [2,7,20–22]. Furthermore, classroom goal orientation is a central aspect of the learning environment, and differences between a mastery climate and a performance climate are likely to be related to school stress [26,27]. A mastery climate emphasizes real learning, understanding and personal improvement, and a high level of mastery climate was associated with lower school stress [2]. A performance climate emphasizes competition and performance relative to others as a measure of success, and a high level of performance climate was associated with higher school stress [2].

Variations in school stress between school classes is an under-researched topic. Students are nested within classes, and one could expect variations between school classes in terms of stress because of differences in the composition of the student groups or contextual factors such as shared cultural, social and physical factors within the class. Multilevel modeling makes it possible to separate the class effects from individual circumstances [28,29]. Prior studies using a multilevel approach investigated topics like students’ mental health, wellbeing and attitudes. Although most of the variation was related to individual factors, some of these studies indicated notable class-level effects (3–10% of the variance) [2,4,28–30].

This multilevel study investigates the relationship between the classroom environment and school stress among adolescents. Moreover, prior research suggests that classroom goal orientation, academic achievement, sex and parental education can be related to schoolwork stress, and it is interesting to study the strength of these relationships and how these variables interact with each other. This study focusses on stress related to schoolwork among students in academic upper secondary schools in Mid-Norway. The research questions asked are: 1. To what degree does students’ schoolwork stress vary between academic classes?; and 2. Are perceptions of classroom goal orientation, academic achievement, sex, and parental education related to schoolwork stress?

## Methods

The data derived from a survey in the southern part of Trøndelag county in Mid-Norway, and the sample can be described as a convenience sample. At the time of the study, the southern part of Trøndelag included 22 upper secondary schools, and 13 of these schools took part in the survey. The survey was administered to eight city schools and five rural schools. All students in the final year of academic studies had the opportunity to participate, and the participants were 18–19 years old. A total of 1955 students were invited to participate in the survey, and 1511 responded, yielding a response rate of 77%. The sample was reasonably representative regarding sex, geography, academic achievement and school size.

The students completed the questionnaires in writing at school, and it took about 30 min to fill out the questionnaire. The students wrote their names on the first page of the survey, and they were not anonymous. An information letter was provided to students before they were asked to complete the survey. The letter explained the main purposes of the study, and that the survey was voluntary. The students who decided not to answer the survey did other school work. The study was ethically approved by the Norwegian Centre for Research Data (approval ID: 42443). The research project was self-funded.

*Schoolwork stress* was measured by three items taken from the study *Young in Oslo* [23], which was inspired by the study *Health Behavior in School-Aged Children* [1]. The items were formulated this way: ‘I get stressed by schoolwork’, ‘I feel exhausted because of schoolwork’, and ‘I have more schoolwork than I can manage’. Response alternatives were on a six-point scale from strongly agree (6) to strongly disagree (1). A sum score was calculated (range 1–6), and Cronbach’s alpha for the scale was good (0.81). Previous analyses also showed satisfactory reliability [23].

*School class variance* was estimated both without and with adjustment for independent variables. The students were from 71 school classes. The number of units needed to give accurate estimates in multilevel models depends on a variety of factors, but there is general agreement among statisticians and researchers that 50 or more units is acceptable in most situations [28,31]. The average number of students from the same class was 21, ranging from 6 to 34 students.

*Sex* was divided between boys and girls.

*Mothers’ education* was divided between students who have a mother with upper secondary school and students who have a mother with bachelor’s degree or higher.

*Academic achievement* was measured as the grade point average of three core subjects (Norwegian, Mathematics and English), ranging from 1 (lowest) to 6 (highest).

*Perception of performance climate* was measured by four slightly modified items from the Patterns of Adaptive Learning Scales [32]; the most important thing for our teachers is to ‘get good grades’, ‘answer questions correctly’, ‘perform well on tests’ and ‘perform well in school’. Response alternatives were on a six-point scale from strongly agree (6) to strongly disagree (1). A sum score was calculated (range 1–6), and Cronbach’s alpha for the scale was good (0.82).

*Perception of mastery climate* was measured by four slightly modified items from the Patterns of Adaptive Learning Scales [32]; the most important thing for our teachers is that ‘we learn new things’, ‘I do as well as I can in the subjects’, ‘I understand subjects instead of only memorizing information’ and ‘it is OK to make mistakes if you learn from them’. Response alternatives were on a six-point scale from strongly agree (6) to strongly disagree (1). A sum score was calculated (range 1–6), and Cronbach’s alpha for the scale was good (0.75).

In addition to individual-level variables, the study also included two class-level variables; *class performance climate* and *class mastery climate*. In order to measure the shared perceptions of goal orientation in school classes, students’ individual assessment of performance climate and mastery climate were aggregated, and a mean score for each class was created [29–31]. The mean scores on the class performance scale varied from 3.0 to 4.4, whereas the mean scores on the class mastery climate varied from 3.8 to 5.1. A major dilemma is that students in the same class can differ considerably in their perceptions of the classroom goal orientation [33]. The standard deviation (SD) is a measure of how dispersed the data is in relation to the mean, and SD can be used to indicate whether students in the same classroom score similar or diverse on performance climate and mastery climate. The average SD for both performance climate and mastery climate divided by classes was 0.9. On a 1–6 scale, 0.9 is considered relatively low, indicating that the scores of the majority of students were clustered around the mean in their class. The range of SD was from 0.6 to 1.3, illustrating high degrees of agreement among students in some classes whereas other classes exhibited more diversity.

The data were analysed using IBM SPSS 27. We used multilevel modeling to analyse the relationship between school class and individual variables with schoolwork stress. A multilevel model was preferred for two reasons. First, schools have a hierarchical structure where students are nested within classes. Multilevel models allow for the investigation of variation in schoolwork stress at both the student level and the class level [28,31]. Second, students within the same class are not independent of each other since they share a common learning environment, interact with the same teachers and belong to the same student group. Previous research has suggested that interactions among students and between students and teachers can impact attitudes, behaviors and emotions, and such interactions can also influence the level of schoolwork stress experienced by individual students within a class [29,30]. The phenomenon of mental health issues, including stress, spreading within social networks is also well-documented in research literature about social contagion [34].

The intraclass correlation coefficient (ICC) describes how strongly units in the same group resemble each other. When multiplied by 100, the ICC gives the percentage of variance attributed to each level. The variance attributable to differences between classes is analysed both without and with adjustment for independent variables.

## Results

Table I displays information about schoolwork stress. The mean and median schoolwork stress score was 4.0. Of all the students who took part, 18% scored lower than 3 on the schoolwork stress scale, 54% scored from 3 and up to 5 and 28% scored 5 and higher. Although the scores of most students in the study clustered around the mean, the SD of 1.2 indicated that there was some diversity in the levels of schoolwork stress among the students in the study. The observed difference in mean scores on schoolwork stress between girls (4.34) and boys (3.56) indicates that, on average, girls report experiencing higher levels of stress related to schoolwork compared with boys.

**Table I.** Schoolwork stress level in academic upper secondary school in the final year: mean with 95% CI, median and SD (n = 1511).

	All	Girl	Boy
Mean schoolwork stress	4.01 (3.95 to 4.07)	4.34 (4.26 to 4.41)	3.56 (3.47 to 3.66)
Median schoolwork stress	4.0	4.33	3.67
SD schoolwork stress	1.22	1.21	1.14

CI, confidence interval; SD, standard deviation

Table II summarizes the characteristics of the participating students divided by sex. The sample comprised 42% boys and 58% girls. 45% of the sample had a mother with bachelor’s degree or higher. The average grade in Norwegian, mathematics, and English was 4.2. The average score of mastery climate was 4.3, and the average score of performance climate was 3.6.

**Table II.** Overview of characteristics of respondents.

	Total sample	Girls	Boys
	Percentage	Percentage	Percentage
Sex			
Girls	58		
Boys	42		
Mother’s educational attainment			
Upper secondary school	55	57	53
Bachelor’s degree or higher	45	43	47
	Mean values	Mean values	Mean values
Average grade (scale 1–6)	4.19	4.25	4.09
Mastery climate (scale 1–6)	4.26	4.27	4.25
Performance climate (scale 1–6)	3.64	3.62	3.66

Table III presents the correlation matrix for all variables in the study. As expected, there was a moderate correlation between girls and schoolwork stress (*r* = 0.31). Individual-level and class-level mastery climate was weak and inversely correlated with schoolwork stress, whereas individual-level performance climate was weak and positively correlated with schoolwork stress. Average grade was weak and inversely correlated with schoolwork stress.

**Table III.** Correlation matrix of the variables in the study (Pearson’s *r*, point biserial, phi).

	1.	2.	3.	4.	5.	6.	7.	8.
1. Schoolwork stress	1							
2. Girl	0.31**	1						
3. Mother with high education	–0.04	–0.04	1					
4. Average grade	–0.14**	0.10**	0.21**	1				
5. Mastery climate	–0.20**	–0.02	0.00	0.17**	1			
6. Performance climate	0.12**	–0.03	–0.02	–0.06	0.05	1		
7. Class mastery climate	–0.13**	0.02	0.04	0.14**	0.31**	–0.03	1	
8. Class performance climate	0.06*	–0.08**	–0.06*	–0.02	–0.03	0.30**	–0.10**	1

Significance: **p ⩽ 0.01, *p ⩽ 0.05.

Multicollinearity refers to a high correlation or interdependence between independent variables, and examining the possibility of multicollinearity is especially important considering that some class-level indicators were aggregates of individual-level indicators. There were moderate correlations between mastery climate and class mastery climate (*r* = 0.31) and between performance climate and class performance climate (*r* = 0.30). In addition, there were weak correlations between average grade and mother’s education, mastery climate and class mastery climate. In conclusion, multicollinearity was not a concern.

Table IV presents the results from the multilevel models about schoolwork stress. Variation in schoolwork stress between classes was initially assessed by means of an empty model without independent variables. Most of the variance in schoolwork stress was accounted for by individual-level factors (94%), but there was also a significant school class-level effect (6%). The between-class variance was reduced somewhat in the full model to 5%.

**Table IV.** Results from multilevel modeling on associations between individual-level and class-level variables and schoolwork stress, ICC, estimates of fixed effects and 95% CI.

	Model I (empty)	Model II (full)	
Factors	Estimates	Estimates	95% CI
ICC % individual-level full model			
ICC % school class-level full model			
Intercept		5.66**	4.02 to 7.32
Girl		0.81**	0.69 to 0.93
Mother with bachelor degree or higher		0.01	–0.11 to 0.13
Average grade		–0.25**	–0.32 to −0.17
Mastery climate		–0.20**	–0.26 to −0.13
Performance climate		0.12**	0.06 to 0.18
Class mastery climate		–0.29*	–0.56 to −0.01
Class performance climate		0.14	–0.17 to 0.44
ICC % individual-level	94	95	
ICC % school class-level	6	5	

Significance: **p ⩽ 0.01, *p ⩽ 0.05.

Reference categories; boy; mother with upper secondary school education.

CI, confidence interval; ICC, interclass correlation coefficient.

Girls had a higher score on schoolwork stress compared with boys (*p* < 0.01). Students with a high average grade reported less schoolwork stress compared with students with low grades (*p* < 0.01). Mastery climate was linked to lower schoolwork stress (*p* < 0.01), whereas performance climate was linked with higher schoolwork stress (*p* < 0.01). Moreover, students attending classes with a higher mean score on mastery climate reported less schoolwork stress (*p* < 0.05). Mother with bachelor’s degree or higher and class performance climate were found to be not statistically significant in relation to schoolwork stress.

## Discussion

As expected, the level of schoolwork stress among the participating students was quite high, and about half of the students had a score 4 or higher (on the 1–6 scale). Prior studies showed that students from academic studies experience a higher workload and more school stress than students in vocational studies [2,7,25]. Moreover, the students in our study were in their final year in upper secondary school, and they may feel pressure to do well in subjects and examinations to achieve a certain grade point average to gain admission to their desired university program [19,25]. Previous qualitative studies found that school was a major source of stress. One aspect was daily school demands with a heavy workload and the other aspect was future-oriented concerns about education and pursuing a particular career path that aligns with the student’s aspirations [23–25].

Individuals may have different resilience levels and coping strategies, and this may relate to how schoolwork stress is reported. Avoidance and passive strategies are likely to impact learning negatively and increase school stress, whereas active coping strategies such as seeking social support, planning and engaging in problem-solving are associated with better academic performance and less school stress [6,17,35]. Thus, students using active coping strategies may turn stress to something positive, and they are also less likely to have high scores on the three negative items to measure schoolwork stress.

The main contribution of this study is the finding that schoolwork stress varies significantly among classes in upper secondary school. While there has been extensive research on stress among students, the application of a multilevel approach specifically to the study of schoolwork stress is relatively novel [10,37]. The notable class-level effect found on schoolwork stress aligned with previous research using a multilevel approach on mental health, attitudes and academic achievement [4,10,13,28–30]. The school culture, class composition, teaching approaches, classroom environment and peer influences can all contribute to variations in schoolwork stress between classes.

As regards peer influences, prior studies on emotional and social contagion have demonstrated that various behaviors and preferences, such as smoking habits, music and movie tastes, emotions like happiness and loneliness, as well as attitudes, can cluster and spread within a group [34]. Likewise, the stress levels of one student could have the potential to influence the stress levels of others within the same class or social group, especially among girls. There are several ways that girls influence each other and exaggerate school-related stress: girls can be influenced by the emotional states of their peers, social comparison can lead to increased stress if girls perceive themselves to be falling behind or not meeting certain expectations compared with their peers, and the pressure to outperform peers or meet certain standards can lead to heightened school stress [17–19,23,24,36].

Another point as regards variations in schoolwork stress is that students in the same class share teaching staff and classroom goal orientation. At the individual level, a mastery climate was associated with less schoolwork stress and a performance climate was associated with higher schoolwork stress. Students’ individual assessments of performance climate and mastery climate were aggregated to the class level. Students attending classes with a higher mean score on mastery climate reported less schoolwork stress, whereas the study did not detect a relation between class performance climate and schoolwork stress.

Studies have consistently shown that adolescents who perceive their classroom as having a mastery climate experience less stress and anxiety related to schoolwork [2,26,27]. This is likely because a mastery climate encourages students to focus on their own competence development and understanding of the material being taught, fostering autonomy and a sense of control over their academic progress, both of which are important for reducing stress and promoting wellbeing. In contrast, a performance climate places greater emphasis on competing with others and achievement outcomes [26,27]. Adolescents in a performance climate may feel pressure to achieve high grades and may compare themselves unfavourably with their peers, leading to feelings of inadequacy and stress. In addition, a focus on performance can lead to a lack of intrinsic motivation and a focus on external rewards, which can further increase school stress.

As expected, schoolwork stress was much higher among girls than boys, and this result confirmed previous research [3,7,10,19–21,23]. Some psychological explanations for greater schoolwork stress among girls include that girls value schoolwork more, that girls exhibit a stronger desire for academic success and have higher educational aspirations than boys, that girls influence each other and exaggerate school-related stress, that external expectations from parents and family are important for girls, and differences in coping strategies between girls and boys [7,13,21,23]. Other explanations consider structural and societal factors, highlighting the differential role of education for women and men in the labour market. With increasing female labour force participation and delayed family formation, investing in education has become increasingly vital for women. The significant overrepresentation of women in higher education may contribute to the pressure to excel in school felt by girls. Women may also be more reliant on formal educational qualifications to overcome gender-related barriers in the workforce and to access high-paying jobs and sectors, traditionally dominated by men [36].

In line with prior studies, students with low grades reported higher schoolwork stress [3,6,12]. Prior studies showed mixed results with regard to connections between parental education and school stress [6,7,18], and this study did not find an association between mother’s education level and schoolwork stress.

## Conclusions

The findings of this study about schoolwork stress point to the importance of the school class, and the crucial role that teachers and peers play as regards to schoolwork stress. The study has some limitations. First, the sample was from a specific Norwegian county and generalizing results to other populations should be done cautiously. Second, cross-sectional studies have limitations when it comes to establishing causal relationships and understanding the dynamic nature of schoolwork stress over time. Thus, the research questions asked in this paper are purely explorative. Third, stress is a subjective experience that manifests in emotional and psychosomatic symptoms, and it is valuable to assess schoolwork stress using self-reported indicators. However, it is important to acknowledge the limitations associated with individual interpretations, social desirability bias, and potential challenges in accurately recalling past experiences. Fourth, the composite measure of schoolwork stress showed satisfactory reliability, but it included just three negative items and captured a small part of the phenomenon schoolwork stress. Fifth, when assessing differences between boys and girls it is important to consider that boys may under-report their schoolwork stress levels if they feel that admitting to high levels of stress would make them appear weak or unable to cope. Sixth, students in the same classroom scored quite similarly on goal orientation variables in most classes, but the observed differences in students’ perceptions of goal orientation within certain classes raise some concerns about the validity of the mastery scale and the performance scale. Finally, schoolwork stress is influenced by a complex interplay of multiple factors that were not included in the study. The inclusion of personal characteristics, individual coping strategies, school culture, class composition, parental support and expectations and many more, may have contributed to greater understanding of variations in schoolwork stress between classes.

There is still much more to learn about school stress in upper secondary school. Further research with a qualitative design can help to shed light on the reasons for variations in school stress between school classes and various student groups. Quantitative and qualitative studies with longitudinal designs could capture changes in school stress over time and investigate causal relationships more rigorously. Triangulating data from multiple sources and employing mixed-methods approaches can provide a stronger basis for understanding causality and capturing a more comprehensive picture of school stress. A final point is that there is much more research on distress than eustress, and the research environment could benefit from more studies on how stress can be beneficial.

Schoolwork stress can have significant effects on students mental health, well-being, and academic achievement. The difference in stress levels between girls and boys point out the need for gender-differentiated approaches in addressing schoolwork stress. The significance of the school class, teachers, and peers in relation to schoolwork stress highlights the importance of fostering a positive and supportive classroom climate. Several systematic literature reviews conclude that school-based mental health interventions could be effective in improving adolescents mental health, including stress [38].

Health and life skills was recently introduced as an interdisciplinary topic at all levels of the education system in Norway, and this universal initiative reflects a commitment to promote health promotion and wellbeing in school. There are many challenges associated with implementing large-scale school curricular changes, such as resistance from various stakeholders, resource constraints, cultural and contextual variations between schools, concerns about teaching methods and how to integrate the topic into the curriculum. Still, we hope that the integration of health-related topics in the curriculum has the potential to contribute to the overall health of adolescents and have a positive impact on school stress.
